# DistMap: A Toolkit for Distributed Short Read Mapping on a Hadoop Cluster

**DOI:** 10.1371/journal.pone.0072614

**Published:** 2013-08-23

**Authors:** Ram Vinay Pandey, Christian Schlötterer

**Affiliations:** Institut für Populationsgenetik, Vetmeduni Vienna, Vienna, Austria; University of Nevada School of Medicine, United States of America

## Abstract

With the rapid and steady increase of next generation sequencing data output, the mapping of short reads has become a major data analysis bottleneck. On a single computer, it can take several days to map the vast quantity of reads produced from a single Illumina HiSeq lane. In an attempt to ameliorate this bottleneck we present a new tool, DistMap - a modular, scalable and integrated workflow to map reads in the Hadoop distributed computing framework. DistMap is easy to use, currently supports nine different short read mapping tools and can be run on all Unix-based operating systems. It accepts reads in FASTQ format as input and provides mapped reads in a SAM/BAM format. DistMap supports both paired-end and single-end reads thereby allowing the mapping of read data produced by different sequencing platforms. DistMap is available from http://code.google.com/p/distmap/

## Introduction

Next Generation Sequencing (NGS) technologies have revolutionized biological research by producing an unprecedented amount of data in a cost effective manner. The rapid progress in NGS technology, however, has resulted in a subsequent increase in data volume that currently outpaces advances in computational power [1]. Hence there is a growing need for new software solutions that minimize the impact of increasing data volume on workflow duration. The first step in nearly all NGS workflows is the mapping of short sequence reads to a reference genome. As it may take several days to map NGS read data on a single computer, mapping is a potential bottleneck for most workflows. For this reason tools and software which support distributed computing are a powerful means to expedite mapping and the subsequent workflow.

At present several tools exist which support distributed mapping, each having specificities which may limit more general usage. For instance, Crossbow [2], Eoulsan [3] and FX [4], are designed for specific types of analyses, do not produce mapping output in SAM/BAM [5] format, and only support paired-end mapping. Similarly, SEAL [6] only supports paired-end BWA mapping using a sinlge version (0.5.8c), and is restricted to Linux operating systems. Other virtual machine workflows include Bio-Linux [7], Cloud Bio-Linux [8] and Galaxy [9]. Bio-Linux and Cloud Bio-Linux include a collection of useful bioinformatics tools, and the later has integrated MapReduce tools such as Crossbow [2], CloudBurst [10], SEAL [6] and bcbio-nextgen [11] but they have restrictions on mapper version and do not produce an output in SAM/BAM format. Galaxy [9] is another powerful workflow system but supports only BWA and bowtie mapping and imposes version restrictions for both the mapper and the reference sequence.

Here we present a new tool, DistMap, a modular, scalable and user-friendly workflow, which facilitates the mapping of short reads on a Hadoop cluster [14]. DistMap supports nine different mappers – the most available in a single distributed mapping tool at present – and cover a wide range of NGS applications. Furthermore, DistMap allows mapping of paired-end and single-end reads from different sequencing platforms and produces mapping output in BAM/SAM file format, such that it can be used in conjunction with distributed downstream analytical tools such as GATK [12], and Hadoop-BAM [13]. Finally, the workflow has been developed specifically for adoption by non-expert users who wish to benefit from distributed NGS read mapping.

## Results and Discussion

**Figure 1:**
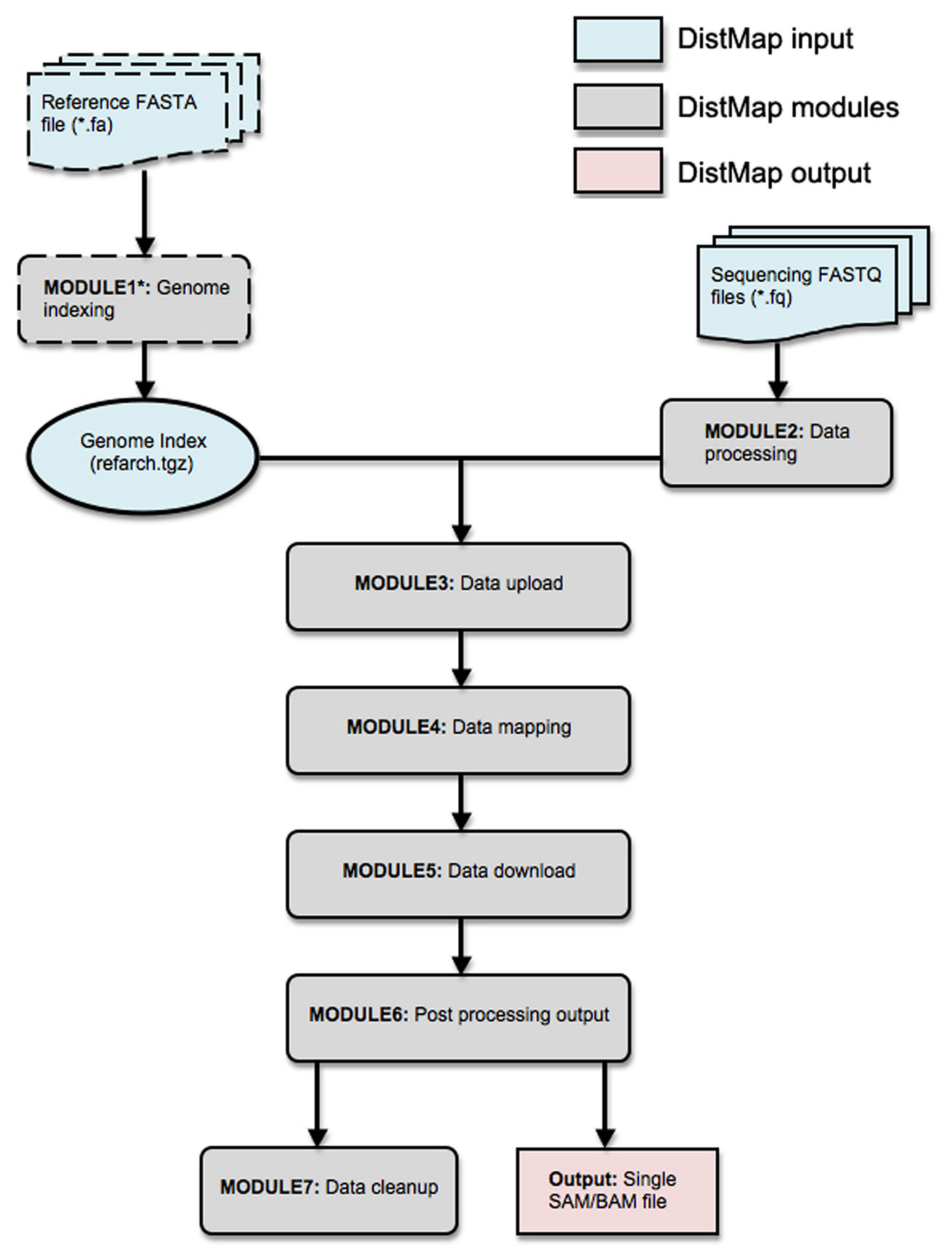
The workflow of DistMap. The DistMap workflow has 7 modules. Modul 1 is to index reference fasta file. Module 2-6 are mandetory for each new FASTQ files. The DistMap entire workflow can be executed at once and if required each module can be executed one by one.

DistMap provides an integrated workflow for short read mapping against a user-specified reference genome. The whole workflow can be run with a single Perl command. This workflow is equipped with various customized parameters and provides detailed guidelines for its implementation. All components of DistMap and their inputs have been summarized in Figure 1. An overview of the key features are described below.

### An integrated workflow

DistMap provides a complete workflow that includes several modules. Each module has a start and stop point (shown in Figure 1). The user is provided with the flexibility to either execute the whole workflow or to execute individual modules one after another.

### Job Scheduler support

DistMap is the only workflow, which supports the different job schedulers currently available for a Hadoop cluster. It supports three schedulers 1) FIFO [14] 2) Fair scheduler [15] and 3) Capacity scheduler [16]. The user can specify the scheduler information with the option -- ***hadoop-scheduler*** within the Perl command.

### Custom queue and pool support

DistMap is designed to manage the short read mapping on a large scale, whereby many custom queues and pools are available within the Hadoop cluster. Unlike other distributed mapping tools which only utilize a default queue, DistMap can also use customized queues and pools to run multiple mapping jobs in parallel. The queue assignment can be done with the option ***–queue-name*** to the Perl command.

### Custom job prioritization support

DistMap supports a top-level job prioritization. The user can directly set job priority via the DistMap command line with the option ***–job-priority***. Five priority levels are currently supported: VERY_LOW, LOW, NORMAL, HIGH and VERY_HIGH.

### Mapper flexibility

The current version of DistMap (v 1.0) supports nine different mappers, covering a broad spectrum of NGS applications. Table 1 provides the corresponding version of all mapping software that has been used for testing. DistMap does not impose a version restriction for any mapper, whereby the user can download and compile any version for direct implementation within their specific workflow.

**Table 1:** List of mappers supported in current version of DistMap.

**Mapper supported in DistMap**	**Version tested**	**Application**
BWA	0.5.8c	Short read mapping
bowtie	0.12.7	Short read mapping
Bowtie2	2.0.6	Short read mapping
GSNAP	2012-07-20	RNA-Seq Alignment DNA methylation
SOAP	2.20	Short read mapping
STAR	2.2.0c	RNA-Seq Alignment
Bismark	v 0.7.7	Bisulfite mapping
BSMAP	2.73	Bisulfite mapping
TopHat	2.0.6	RNA-Seq mapping

Nine short read mappers for a wide range of NGS applications are supported. DistMap can use any version of these mappers.

### Flexibility and transparency

DistMap imposes no restriction on the assembly version of the reference genome for mapping. Since DistMap does genome indexing itself during the execution of the workflow it is possible to map short reads with any reference genome assembly. DistMap collects all input files and parameters from a local computer and returns the final output to a local output directory as a single SAM or BAM file. There is no need to install the DistMap source code or mapper executables on all working nodes. The entire DistMap workflow can be run at once or in step-by-step fashion, such that the user can start from any step in the workflow. DistMap supports paired-end, single-end and mixed mapping of FASTQ formatted reads produced from various sequencing platforms. DistMap archives the genome index as a *. tgz file in the local output directory such that it can be re-used in subsequent mapping and thereby avoids unnecessarily re-indexing of the same genomes.

### Scalability

DistMap has no restriction on NGS data handling; it is specifically designed to map Gigabytes or Terabytes of sequencing data with a single command. The speed of DistMap scales with cluster size. Several different datasets ranging in size were used to test the scalability of the DistMap workflow on a Hadoop cluster (see Table 2)

**Table 2:** DistMap evaluation input datasets.

**Number of read pairs (million)**	**Size (GB)**	**Read length (bp)**
5	2.46	100
50	24.7	100
100	49.4	100
200	98.82	100
500	247.04	100

These NGS data were generated from Illumina HiSeq sequencer. 100bp paired-end reads from *Drosophila melanogaster* genomes.

### DistMap evaluation

To evaluate the performance of DistMap we used paired-end genomic reads of 2x100bp from a *Drosophila melanogaster* pooled sequencing project [17] (see Table 2 for further information). The Hadoop cluster (version 1.0.3) consisted of 13 nodes running Mac OSX 10.6.8. For each node 10 CPUs, 32 GB RAM and 1TB SATA hard disk space was made available. All computer nodes were connected via Gigabit Ethernet.

**Figure 2:**
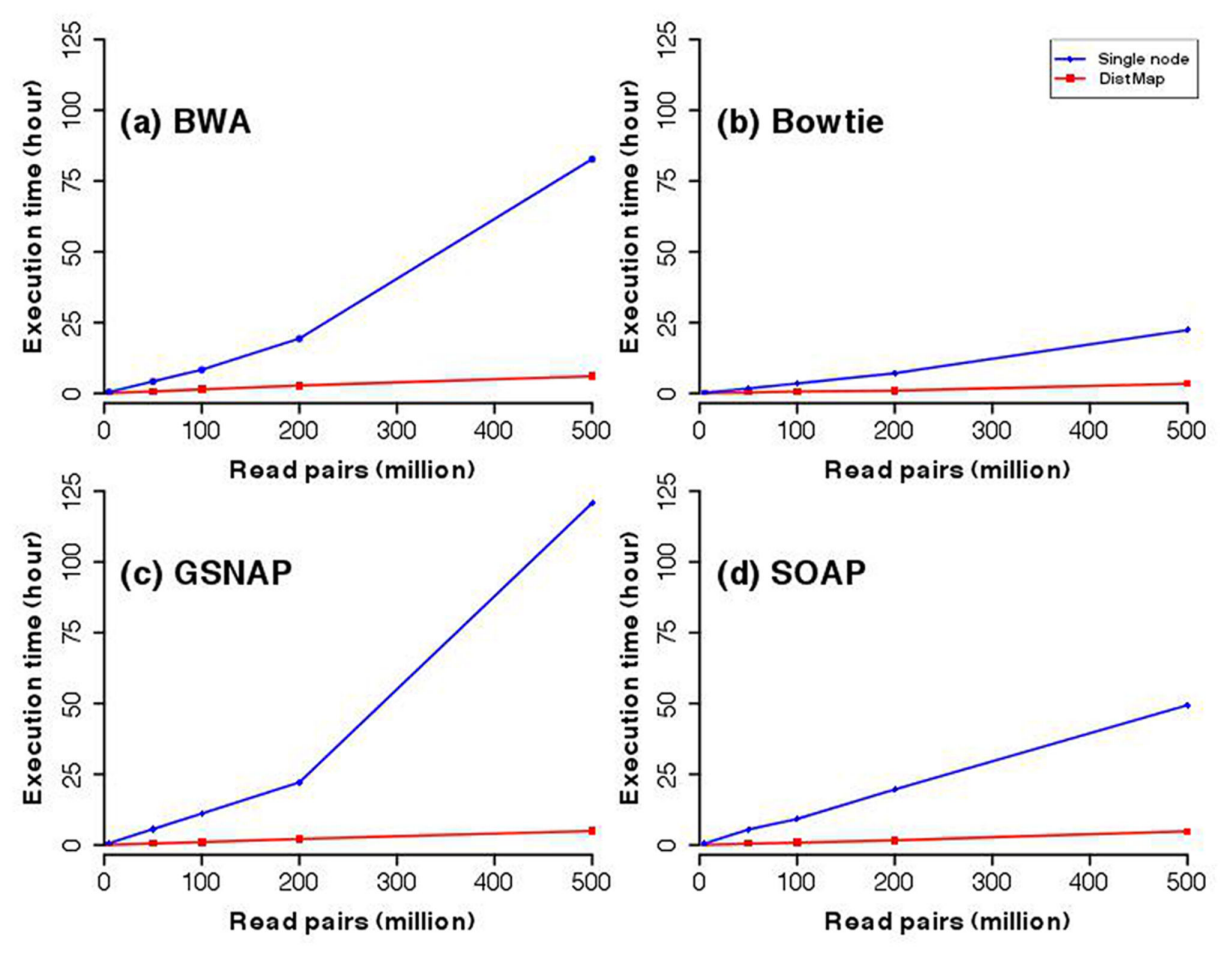
Evaluation of DistMap execution time with increase of data size. Execution times were measured for different mappers using DistMap (red line) and compared to a single node (blue line). Datasets of different size (5, 50, 100, 200 and 500 million read pairs) from different pool-Seq experiments were used to estimate the scalability of DistMap. The hardware configuration of the single node was a Mac OSX 10.6.8 computer with 10 CPU, 32 GB RAM and 1TB Disk space available for mapping. The Hadoop cluster consists of 13 worker nodes with the same configuration. (**a**) BWA mapping (**b**) bowtie mapping, (**c**) GSNAP mapping, (**d**) mapping. All mappers were run with the same default parameters and datasets.

We evaluated the performance of DistMap by comparing the execution time of four different mappers (BWA, bowtie, GSNAP and SOAP) for multiple datasets ranging in size, each time using a single worker node and a fixed set of parameters (see Figure 2 and Table 3). We found that DistMap execution time scales almost linearly with increase of input size, whereas single node execution increased exponentially. In particular, as data size increases beyond 500 million reads, DistMap had between 20-fold to 80-fold reduction in processing time relative to a single computer for the mappers tested. We reason that the non-linear scaling observed for the single node results from non-parallelized steps in the mapping procedure. For example, BWA only uses a single processor for the *sampe/samse* module, regardless of the number of processors on that computer. In contrast, DistMap can make use of all cluster nodes and their processors by splitting the data into subsets, which are later reassembled. Thus, by distributing mapping across several computers, DistMap avoids probable workflow bottlenecks caused by mapping on a single computer.

**Table 3:** Comparison of running time in hours of different mappers **on a single node with 10 cores and DistMap running on 13 nodes**.

**Number of read pairs (million)**	**Mapper**	**DistMap time (hour, 13 nodes)**	**Mapper time (hour, 1 node)**
5	BWA	0.12	0.47
50	BWA	0.70	4.22
100	BWA	1.40	8.33
200	BWA	2.78	19.35
500	BWA	6.05	82.73*
5	bowtie	0.10	0.18
50	bowtie	0.30	1.77
100	bowtie	0.68	3.50
200	bowtie	0.97	7.10
500	bowtie	3.43	22.38
5	GSNAP	0.1	0.63
50	GSNAP	0.57	5.62
100	GSNAP	1.07	11.12
200	GSNAP	2.17	22.13
500	GSNAP	4.97	120.75*
5	SOAP	0.08	0.52
50	SOAP	0.47	5.48
100	SOAP	0.87	9.2
200	SOAP	1.68	19.63
500	SOAP	4.88	49.4

* Indicates cases where DistMap is faster than separate mapping on 13 individual nodes

### Reproducibility and accuracy

We estimated the reproducibility, fault tolerance and data security of DistMap by mapping 5 million read pairs multiple times for each of the BWA, bowtie, GSNAP and SOAP mappers. Fault tolerance and data security were specifically tested by randomly deactivating nodes during an active mapping job. Even with random node deactivation, however, mapping results were found to be identical across the five independent runs for each mapper. Indeed, because DistMap makes a minimum of three copies of each data block, each distributed to a different node, data loss is highly improbable.

### Comparison with existing tools

DistMap can be run on any Unix system as a single Perl command and has many user-friendly features, which enable both advanced and non-expert users to map NGS reads using distributed computing. The workflow can be executed as a whole or individual modules can be executed one after another. A comparison of the most important features of DistMap to other available tools is given in Table 4.

**Table 4:** Comparison of various features of DistMap and other tools for distributed mapping.

**Features**	**Crossbow v 1.2.0**	**SEAL v 0.1.0**	**Eoulsan v 1.1.6**	**FX v 1.0.4**	**DistMap v 1.0**	**Galaxy**
SAM output	no	no	no	no	yes	yes
BAM output	no	no	no	no	yes	yes
Pair-end Mapping	yes	yes	yes	yes	yes	yes
Single-end Mapping	yes	no	no	no	yes	yes
Bisulfite Mapping	no	no	no	no	yes	no
Installation required	yes	yes	yes	yes	no	yes
Dependency	yes	yes	yes	yes	no	yes
Operating system	All Unix	Only Linux	Only Linux	All OS	All Unix	All OS
Mappers (complied)	bowtie	BWA	BWA, bowtie, SOAP, GSNAP	GSNAP	BWA, bowtie, Bowtie2, GSNAP, SOAP, STAR, TopHat, TopHat2, Bismark, BSMAP	bowtie, BWA
Mapper version dependency	no	yes	yes	yes	no	yes
Reference sequence version dependency	no	no	no	no	no	yes
Custom queue/pool support	no	no	no	no	yes	no
Fair scheduler and Capacity scheduler	no	no	no	no	yes	no

## Materials and Methods

### Implementation

The DistMap was developed in Perl 5.8.8 [18] to map millions of short reads produced from a single NGS experiment in a distributed manner. The mapping module of DistMap is based on the MapReduce programming algorithm [19], which runs on the Hadoop cluster. There is no dependency required to use DistMap except a working Hadoop cluster. The minimum input requirements to run DistMap on any Unix operating system are (1) executables of the required mapping software, (2) MergeSamFiles.jar and SortSam.jar, two jar files from PICARD [20], (3) access to a Hadoop cluster, (4) FASTQ formatted reads (paired-end, single end or mixed) and (5) a FASTA formatted reference genome.

### Hadoop and MapReduce

Hadoop is an open source software framework, which can be installed and run on commodity computers and enables large-scale distributed data analysis. There are two components of Hadoop (1) a fault-tolerant and robust Hadoop Distributed File System (HDFS) and (2) MapReduce: a java-based API, which enables parallel computing across all nodes of a cluster.

Currently nine different mappers are supported in DistMap: BWA [21], bowtie [22], Bowtie2 [23], GSNAP [24], SOAP [25], STAR [26], Bismark [27], BSMAP [28] and TopHat [29], thereby supporting a wide range of biological applications (Table 1). While TopHat could be run on DistMap, we do not recommend this since the distributed mapping interferes with the identification of splice sites by TopHat. DistMap supports two different implementations of customized queuing systems: Capacity scheduler [26] and pool: Fair scheduler [27].

### DistMap components

The DistMap workflow consists of seven main modules which can be executed either end-to-end by a single command, or each module can be executed by giving appropriate command line flags. The whole workflow is summarized in Figure 1.

### DistMap input and output

DistMap workflow takes the input from a local computer, performs the mapping of the reads on a Hadoop cluster, and stores the final output file on the local computer. No direct interaction between the user and the Hadoop cluster is needed. DistMap requires reference sequence data in FASTA format and short read data in FASTQ format. If the user has to map many datasets in a single DistMap run then it can be done via command line by using the option ***–input*** multiple times. The final output of DistMap is a single SAM or BAM file without any filtering. The user can request either a SAM or a BAM output file by using the DistMap command line option ***–output-format***.

### Availability, installation and usage

DistMap is an open-source tool and is freely available for all researchers. The source code of the DistMap can be downloaded from http://distmap.googlecode.com/files/DistMap_v1.0.tar.gz.

The user manual of DistMap is available on http://code.google.com/p/distmap/wiki/Manual.

Since DistMap was developed with easy use for non-expert researchers in mind, we provide a step-by-step guide to set up a Hadoop cluster on Linux computers http://code.google.com/p/distmap/wiki/SetupHadoopLinux and on Macintosh computers http://code.google.com/p/distmap/wiki/SetupHadoopMacintosh.

## Conclusions

DistMap is a user-friendly, modular, and integrated workflow for distributed mapping of NGS-generated short reads on a Hadoop cluster. Since in most NGS applications mapping is an essential and highly time intensive step, we believe that DistMap will be greatly expedite this process and the subsequent workflow. In comparison to other tools, DistMap stands out for its generality and flexibility, supporting nine different mappers that facilitate a range of different NGS-based analyses. The availability of multiple mappers means that DistMap can be readily integrated into many existing workflows without having to incorporate a new mapper. Similarly, the SAM/BAM output format was chosen to be compatible with the most widely used downstream analytical tools. Furthermore, unlike other distributed read mapping tools, DistMap supports customized queuing, multiple job schedulers, and job prioritization within a queue. Finally, DistMap was built to be accessible to novice and advanced users alike, being executed via a single Perl command. To help facilitate its use an extensive user manual and the step-by-step instructions for setting up a Hadoop cluster on Macintosh or Linux computers are provided alongside the source code.
